# Pleiotropic Meta-Analyses of Longitudinal Studies Discover Novel Genetic Variants Associated with Age-Related Diseases

**DOI:** 10.3389/fgene.2016.00179

**Published:** 2016-10-13

**Authors:** Liang He, Yelena Kernogitski, Irina Kulminskaya, Yury Loika, Konstantin G. Arbeev, Elena Loiko, Olivia Bagley, Matt Duan, Arseniy Yashkin, Svetlana V. Ukraintseva, Mikhail Kovtun, Anatoliy I. Yashin, Alexander M. Kulminski

**Affiliations:** Biodemography of Aging Research Unit, Social Science Research Institute, Duke University Durham, NC, USA

**Keywords:** pleiotropic analysis, age-related traits, CVDs, genetic association study, mediation analysis, age-dependent effects

## Abstract

Age-related diseases may result from shared biological mechanisms in intrinsic processes of aging. Genetic effects on age-related diseases are often modulated by environmental factors due to their little contribution to fitness or are mediated through certain endophenotypes. Identification of genetic variants with pleiotropic effects on both common complex diseases and endophenotypes may reveal potential conflicting evolutionary pressures and deliver new insights into shared genetic contribution to healthspan and lifespan. Here, we performed pleiotropic meta-analyses of genetic variants using five NIH-funded datasets by integrating univariate summary statistics for age-related diseases and endophenotypes. We investigated three groups of traits: (1) endophenotypes such as blood glucose, blood pressure, lipids, hematocrit, and body mass index, (2) time-to-event outcomes such as the age-at-onset of diabetes mellitus (DM), cancer, cardiovascular diseases (CVDs) and neurodegenerative diseases (NDs), and (3) both combined. In addition to replicating previous findings, we identify seven novel genome-wide significant loci (< 5e-08), out of which five are low-frequency variants. Specifically, from Group 2, we find rs7632505 on 3q21.1 in *SEMA5B*, rs460976 on 21q22.3 (1 kb from *TMPRSS2*) and rs12420422 on 11q24.1 predominantly associated with a variety of CVDs, rs4905014 in *ITPK1* associated with stroke and heart failure, rs7081476 on 10p12.1 in *ANKRD26* associated with multiple diseases including DM, CVDs, and NDs. From Group 3, we find rs8082812 on 18p11.22 and rs1869717 on 4q31.3 associated with both endophenotypes and CVDs. Our follow-up analyses show that rs7632505, rs4905014, and rs8082812 have age-dependent effects on coronary heart disease or stroke. Functional annotation suggests that most of these SNPs are within regulatory regions or DNase clusters and in linkage disequilibrium with expression quantitative trait loci, implying their potential regulatory influence on the expression of nearby genes. Our mediation analyses suggest that the effects of some SNPs are mediated by specific endophenotypes. In conclusion, these findings indicate that loci with pleiotropic effects on age-related disorders tend to be enriched in genes involved in underlying mechanisms potentially related to nervous, cardiovascular and immune system functions, stress resistance, inflammation, ion channels and hematopoiesis, supporting the hypothesis of shared pathological role of infection, and inflammation in chronic age-related diseases.

## Introduction

Common age-related diseases including cancer, cardiovascular diseases (CVDs), diabetes mellitus (DM), and neurodegenerative diseases (NDs) such as Alzheimer's disease (AD) and dementia are major contributors to morbidity and mortality at old age. The underlying hypothesis in gerontology posits that these devastating diseases share some common mechanisms in intrinsic processes of aging that remain to be determined (Kaeberlein et al., 2015). Identifying genetic variants associated with multiple age-related diseases (i.e., pleiotropic effects) is a fundamental step toward understanding the genetic contribution to age-related processes. Numerous loci have been identified to show association across distinct diseases such as immune-related disorders, cancer and NDs, highlighting the common underlying biological pathways in the etiology of these complex diseases (Sivakumaran et al., 2011). For example, variants in *APOE* significantly associated with longevity and lifespan also confer risk of CVDs, AD and cancer (Ewbank, 2002; Christensen et al., 2006; Brooks-Wilson, 2013; Kulminski et al., 2014, 2015a). A putative loss of function variant in *PTPN22* has been found to reduce the risk of Crohn's disease but to increase the risk of rheumatoid arthritis and DM (Plenge et al., 2005; Todd et al., 2007; Barrett et al., 2008). On the other hand, evidence suggests that many age-related diseases are associated with a variety of intermediate phenotypes (also called endophenotypes, i.e., a hereditary characteristic that normally relates to some condition but is not a direct symptom *per se*) such as body mass index (BMI), lipids, etc. It has been noted from previous genome-wide association studies (GWAS) that different diseases and endophenotypes can be linked by shared genetic variants (Li et al., 2014). These observations demonstrate the clinical benefits from characterization of shared genetic causes that have pleiotropic effects on different phenotypes related to aging. Thus, there has been an increasing interest in jointly interrogating the cross-phenotype association of genetic variants.

Pleiotropic analysis generally provide more information on the common underlying biological mechanism and guidance of further functional studies (Solovieff et al., 2013; Price et al., 2015). In many cases, considering multiple phenotypes together boosts statistical power by aggregating marginal effects on individual phenotypes. Single nucleotide polymorphisms (SNPs) with moderate causal effects on each phenotype may happen not to reach significance when interrogating them separately. Multi-trait analysis has been demonstrated to yield enhanced statistical power for detecting the quantitative trait loci (QTL) that are associated with multiple traits (Korol et al., 2001). Moreover, once genetic pleiotropy is identified, it is of interest to further discriminate between a biological and mediated pleiotropy (Solovieff et al., 2013). The former refers to a direct independent biological effect of a variant on more than one phenotype, while the latter refers to a situation in which the effect on one phenotype is mediated by another phenotype so that the variant is indirectly associated with the second. Given the identified genetic variant having pleiotropic effect on a pair of phenotypes, causal inference under certain specific assumptions can provide insights into potential causal pathways and contribute to characterizing the underlying biological mechanisms. For example, given the causal relationship between low-density lipoprotein (LDL) and myocardial infarction (MI), conducting mediation analysis on a variant associated with both can provide evidence of which pathological pathway the variant is probably involved in. Finally, it should be noted that although previous GWAS following the traditional biomedical strategy have attempted to discover genes conferring risks of age-related diseases, the SNPs associated with them in general are not subject to direct evolutionary selection as these diseases occur during the post-reproductive period so that these SNPs hardly contribute to fitness (Nesse et al., 2012). Therefore, any effect of such SNPs on the diseases is probably mediated through their effects on certain endophenotypes or is modulated by environmental factors (e.g., the grandmother effect; Lahdenperä et al., 2004). Consequently, effects of relevant variants are often found to be environment-specific and to have different temporal patterns (De Benedictis and Franceschi, 2006; Kulminski, 2013; Yashin et al., 2015). In this case, pleiotropic analysis combined with follow-up mediation analysis and annotation with various expression and regulatory database resources can serve as a substantive tool to elucidate the potential mediator and causality behind the association between SNPs and age-related diseases, which may explain inconsistent genetic effects in different populations even in case of populations of the same ancestry and perfectly harmonized phenotypes (Kulminski, 2013; Yashin et al., 2015).

Over the past decade, GWAS have seen unprecedented success; however, most studies focused on individual phenotypes. While approximately >10,000 genome-wide significant SNPs have been identified to be associated with various phenotypes, studies for identifying genetic pleiotropy of age-related diseases and endophenotypes still lag behind. In recent years, a handful of statistical frameworks based on multivariate or univariate approaches have been proposed (Solovieff et al., 2013). Multivariate models are one of the extensively adopted approaches that have been proposed to deal with multiple phenotypes in a unified framework (O'Reilly et al., 2012; Shriner, 2012). Multivariate approaches take into account phenotypic correlation but often have some limitations in the context of meta-analysis and drawbacks of being incapable of handling phenotypes of different types. For example, it may suffer from reduced statistical power due to removal of subjects with missing values in any phenotype. When searching for the pleiotropy between age-related diseases and endophenotypes, we often face the problem of combining time-to-event outcomes and quantitative or binary traits. In this situation, univariate approaches based on *p*-values (Fisher, 1925; van der Sluis et al., 2013) or summary statistics (Xu et al., 2003; Zhu et al., 2015) exhibit unique advantages in terms of both statistical power and flexibility. In particular, pleiotropic analysis by systematically combining summary statistics based on existing GWAS results that also takes into account phenotype correlation can be a fast and simple solution to this issue (Zhu et al., 2015).

In this study, we aim at identifying SNPs that exhibit effects on multiple endophenotypes and age-related diseases including CVDs, cancer, DM and NDs by performing a pleiotropic meta-analysis based on five National Institutes of Health (NIH)-funded large-scale longitudinal datasets. We use a slightly modified strategy based on (Zhu et al., 2015) to combine summary statistics from univariate association studies that takes into account the correlation between the statistics for the endophenotypes and diseases. For each newly identified variant that passes a pre-specified significance threshold, we explore its biological implication and regulatory role by performing follow-up functional annotation and examining Encyclopedia of DNA Elements (ENCODE) (The ENCODE Project Consortium, 2012) and Genotype-Tissue Expression project (GTEx) (Lonsdale et al., 2013) databases. This helps detect spurious pleiotropy that may result from linkage disequilibrium (LD) with two causal variants in different genes affecting different endophenotypes or diseases (Solovieff et al., 2013). We further conduct age-dependent time-to-event analyses to investigate temporal patterns of the effects. To distinguish two different types of pleiotropy, we perform a follow-up mediation analysis using a marginal structural model. In particular, to provide more insight into the plausible causal pathways, we focus on studying whether or how much the effect of an identified SNP on an associated disease is mediated through a specific endophenotype.

## Materials and methods

### Study cohorts

The phenotypic and genotypic data used in this study were collected from the Atherosclerosis Risk in Communities study (ARIC) (http://www.ncbi.nlm.nih.gov/projects/gap/cgi-bin/study.cgi?study_id=phs000090.v1.p1), the Framingham Heart Study (FHS) (http://www.ncbi.nlm.nih.gov/projects/gap/cgi-bin/study.cgi?study_id=phs000007.v28.p10), the Multi-Ethnic Study of Atherosclerosis (MESA) (http://www.ncbi.nlm.nih.gov/projects/gap/cgi-bin/study.cgi?study_id=phs000209.v2.p1), the Cardiovascular Health Study (CHS) (http://www.ncbi.nlm.nih.gov/projects/gap/cgi-bin/study.cgi?study_id=phs000287.v3.p1), and the Health and Retirement Study (HRS) (http://www.ncbi.nlm.nih.gov/projects/gap/cgi-bin/study.cgi?study_id=phs000428.v1.p1; http://hrsonline.isr.umich.edu/). For FHS, we included the datasets of the FHS Original cohort (FHS cohort 1) and the FHS Offspring cohort (FHS cohort 2). Details of the study design, collection of samples, measurement of phenotypes and genotyping have been described in previous publications [ARIC: (The ARIC investigators, 1989; Sharrett, 1992), FHS: (Splansky et al., 2007; Govindaraju et al., 2008; Cupples et al., 2009), MESA: (Bild et al., 2002), CHS: (Gottdiener et al., 2000), HRS: (Sonnega et al., 2014)]. In each study, we only included non-Hispanic Caucasian subjects. Table 1 summarizes the basic characteristics of the samples and provides a list of the phenotypes we investigated. We included all longitudinal observations of these phenotypes available throughout the follow-up in these studies except HRS in which only one observation was available from the examinations in 2006–2008. The binary outcomes were adopted for the diseases in HRS of which the age-at-onset outcomes were not available. Specifically, we included phenotype measurements from 4 visits in ARIC, 28 visits in the FHS cohort 1, 8 visits in the FHS cohort 2, 5 visits in MESA, and 22 examinations in CHS. For FHS, we performed the analyses for the two cohorts separately to account for potential generation confounders.

**Table 1:** **Basic characteristics of the samples, endophenotypes, and diseases in the six cohorts included in the analyses**.

**Study**	**Total observations**	**Sample size^a^**	**Number of families**	**Age^b^**	**Female (%)**	**Endophenotype^c^**	**Diseases or death^d^**
ARIC	38,472	9618	9093^e^	54.32	52.99	VR, BG, BMI, SBP, DBP, HDLC, TG, TC, HC	AF, Cancer, CHD, DM, HF, Stroke, Death
FHS C1	30,212	1079	675	37.68	61.08	VR, BG, BMI, SBP, DBP, HDLC, TG, TC, HC	Cancer, CHD, DM, ND, HF, IC, Stroke, Death
FHS C2	28,120	3515	1191	35.42	52.83	VR, BG, BMI, SBP, DBP, HDLC, TG, TC	Cancer, CHD, DM, ND, HF, IC, Stroke, Death
MESA	12,275	2455	2421	62.67	52.06	VR, BG, BMI, DBP, HDLC, TG, TC, PP, HbA1c	CHD, CVD, DM, HF, Death
CHS	66,946	3043	3043	72.39	60.4	VR, BG, BMI, SBP, DBP, HDLC, TG, TC, HC, Creatinine, CRP, FEV1	AD, Cancer, CHD, CVD, DM, HF, Death
HRS	9704	9704	9643	58.13	57.95	BG, BMI, HDLC, TC, CRP, Cystatin C	Cancer, DM, stroke, HBP, Lung, Psych, Arthr, CHD, Death

a *The sample size corresponds to the number of subjects after exclusion of those with the missing rate >0.05*.

b *Average age of the individuals included at the entry of the follow-up*.

c All endophenotypes included were quantitative traits. SBP was not included in the pleiotropic analysis for MESA as it was highly correlated with PP (r = 0.83) and DBP (r = 0.71) (Figure S4). FVC was not included in the pleiotropic analysis for CHS as it was highly correlated with FEV1 (Figure S5). PP, pulse pressure, HbA1c, Hemoglobin A1c. CRP, C-reactive protein. Log-transformation (100^*^log(.)) was performed for VR, BG, BMI, HDLC, TC, TG, and PP to correct for their distribution skewness.

d *Cancer, DM, Stroke, Lung (lung diseases), Psych (Psychiatric diseases), Arthr (Arthritis), CHD in HRS were binary traits, and the others were time-to-event traits. HBP, IC, and AD refer to hypertension, intermittent claudication, and Alzheimer's diseases, respectively. ND in the FHS cohorts included AD and dementia*.

e *The number of families in ARIC was estimated from a kinship matrix based on the genotype data of the ARIC subjects*.

### Accession numbers

This manuscript was prepared using a limited access dataset obtained though dbGaP (accession numbers phs000007.v22.p8, phs000280.v2.p1, phs000209.v12.p3, phs000287.v3.p1).

### Genotyping, imputation, and quality control

Genotyping of 12,771 ARIC participants (*N* = 9633 whites, *N* = 9618 included) and 8224 MESA participants (*N* = 2686 whites, *N* = 2455 included) was conducted using an Affymetrix 6.0 array (1000 K SNPs). Genotyping of 9167 participants (*N* = 4594 whites included) in FHS was conducted using an Affymetrix 500 K array. Genotyping of 3043 participants in CHS was done using a HumanOmni1-Quad Illumina array. In HRS, ~2.5 M SNPs for 12,507 subjects (*N* = 9736 whites, *N* = 9704 included) were genotyped using an Illumina HumanOmni 2.5 Quad chip. All genotyped SNPs were further filtered for the analysis according to the exclusion criteria of the Hardy-Weinberg equilibrium (HWE) test (*p* < 1e-05) and missing rate (>5%). For the SNPs that were selected as candidates from ARIC (described in the following subsection) and were missing in the other studies, we imputed the genotypes with IMPUTE2 (Howie et al., 2009) according to a 1000 Genomes Project Phase I reference panel. Within each study, those SNPs with an imputation information score < 0.8 were excluded from the analyses.

### Selection of candidate SNPs

To narrow down the candidates and pre-select promising SNPs, we focused on one of the largest studies ARIC, which shared the same genotyping platform with MESA, and reported a wide spectrum of measured relevant phenotypes and diseases. Accordingly, we expected larger enrichment of significant SNPs associated with various age-related diseases and related traits in this study compared to the others. SNPs pre-selected in ARIC have been considered as a starting point for the follow-up meta-analyses to replicate their effects in other studies. We first conducted univariate GWAS of the SNPs with minor allele frequency (MAF)>2% in ARIC for the endophenotypes [ventricular rate (VR), blood glucose (BG), BMI, systolic blood pressure (SBP), diastolic blood pressure (DBP), high-density lipoprotein cholesterol (HDLC), total cholesterol (TC), triglycerides (TG), hematocrit (HC)], the diseases [atrial fibrillation (AF), cancer, coronary heart disease (CHD), DM, heart failure (HF), stroke] and mortality using PLINK software (Purcell et al., 2007). An additive genetic coding model was adopted in all the studies performed at the current and following stages. We computed combined *p*-values across the endophenotypes and diseases using Fisher's method which did not take into account phenotype correlation and thus at this stage produced less stringent results in most cases. The SNPs with a *p*-value exceeding the genome-wide significance threshold (5e-08) were chosen as candidates for the downstream pleiotropic meta-analysis.

### Univariate association analysis

For the selected SNPs, we conducted univariate association analyses separately in each study to obtain summary statistics. The phenotypes and diseases under investigation in each study are listed in Table 1. We adopted a (generalized) linear mixed-effects model with both individual- and family-level random effects to control for family structure and longitudinal observation correlation in ARIC, FHS, and MESA, and with only an individual-level random effect in CHS which consisted of only independent individuals. We adopted a linear regression model for HRS as we only selected cross-sectional measurements and more than 99% of the samples were independent. We adopted marginal Cox proportional hazards (PH) models to analyze the time-to-event phenotypes with robust estimates of variance (Lin and Wei, 1989) for correcting for family structure. We included age and sex as covariates in the analysis of each study. In addition, we included study center as a covariate in ARIC, MESA and CHS, cohort in HRS, and genotyping phase in FHS. We utilized the *lmer* and *lm* functions in the *lme4* R package (Bates et al., 2014) for the quantitative analyses, *glm* for the logistic regression (only for HRS) and the *coxph* function in the *survival* R package (Therneau and Lumley, 2015) for the time-to-event analyses. Log-transformation was performed to correct the skewness of the distribution for certain quantitative phenotypes (Table 1).

### Pleiotropic meta-analysis

**Figure 1:**
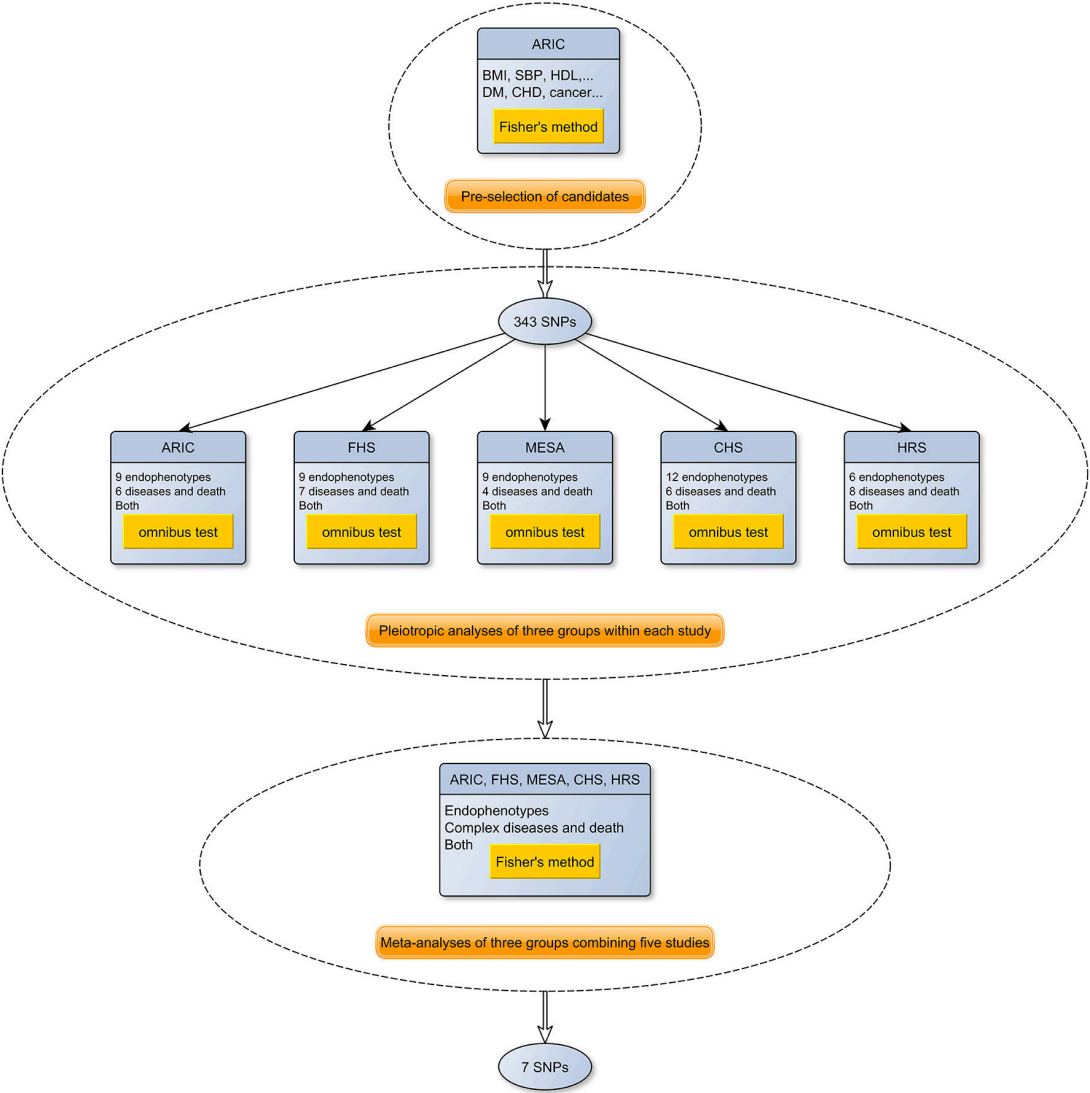
**A flowchart of the meta-analysis of the pleiotropic studies using the five NIH-funded datasets**. As shown in the figure, the meta-analysis consists of three stages. In the first stage, 343 promising candidate SNPs were selected from ARIC. Next, association tests for each endophenotype and disease were performed in each of the five cohorts, and the summary statistics were used to construct three phenome-wide meta-analysis *p*-values (endophenotypes, diseases and both) using the omnibus test. Finally, the *p*-values from each cohort were combined using Fisher's method.

The overall framework of the entire meta-analysis is depicted in the flowchart (Figure 1). Given that Fisher's method does not account for the correlation between the statistics, inflated *p*-values were expected in the pre-selection stage as most of our phenotypes were weakly to modestly correlated (Figures S1–S6). Therefore, we adopted a two-step strategy to compute more accurate meta-analysis combined *p*-values for the selected SNPs. More specifically, suppose that for a certain SNP we observe an estimated effect size ${\hat{\beta }}_{ij}$ together with its standard error ${\hat{\sigma }}_{ij}$ for the phenotype *i* (*i*∈{1, …, *K*_*j*_}) in the study *j (j*∈{1, 2, 3, 4, 5, 6}), where *K*_*j*_ is the number of phenotypes in the study j. In the first step, following the similar spirit of (Xu et al., 2003; Bolormaa et al., 2014; Zhu et al., 2015), we constructed a general omnibus test statistic ${\hat{\text{z}}}_{\text{j}}^{\prime }\Sigma_{\text{j}}^{-\text{1}}{\hat{\text{z}}}_{\text{j}}$ that took into account the correlation of effect sizes between the phenotypes, where ${\hat{\text{z}}}_{\text{j}}={\hat{\beta }}_{\text{j}}\text{/}{\hat{\sigma }}_{\text{j}}$ (${\hat{\beta }}_{j}\;={\left({\hat{\beta }}_{1j},\ldots ,{\hat{\beta }}_{K_{j}j}\right)}^{\prime }$ and ${\hat{\sigma }}_{j}=\left({\hat{\sigma }}_{1j},\ldots ,{\hat{\sigma }}_{K_{j}j}\right)\prime$) and **Σ**_*j*_ is the correlation matrix to be estimated. Thus, under the null hypothesis (**β**_*j*_ = 0) and the assumption of a large sample size, the test statistic approximately follows a chi-square distribution with a degree of freedom (DF) of K_j_

$$\begin{array}{l} {{\hat{\text{z}}}_{\text{j}}}^{\prime }{\Sigma_{\text{j}}}^{-\text{1}}{\hat{\text{z}}}_{\text{j}}\sim \chi_{{\text{K}}_{\text{j}}}^{2}, \end{array}$$

from which we obtained a combined *p*-value *p*_*j*_ in the study *j*. As independent SNPs are recommended for estimating **Σ**_***j***_ (Zhu et al., 2015), we simulated 1000 independent SNPs under HWE based on MAF and estimated the correlation matrix Σ_***j***_ for each study j based on the summary statistics of the simulated SNPs. We checked the multivariate normality assumption for these summary statistics under the null hypothesis using the Henze-Zirkler test (Henze and Zirkler, 1990), and found no evidence of violation of the assumption. The endophenotypes included in the pleiotropic analysis are purported to affect the onset of the complex diseases to some extent. We observed that the majority of the pairwise correlations of the summary statistics from the univariate analysis were lower than 0.3. As combining evidence from highly correlated phenotypes is less informative, within each study, we selected a group of phenotypes (Table 1) to ensure that the correlation between any pair of z-scores was less than 0.7 (The correlation matrices of the statistics in the five datasets are depicted in Figures S1–S6). A *p*-value (e.g., *p*_*j*_) that was smaller than a pre-specified threshold led to the rejection of the null hypothesis, indicating that the SNP was associated with at least one of the phenotypes in the study *j* (i.e., **∃****i**, **β**_**ij**_ **≠** **0**). Then, we computed a *p*-value for the meta-analysis using the Fisher's method in which the samples from the six cohorts were treated as independent (we did not observe evident correlation between the summary statistics under the null hypothesis from the two generations in FHS). Thus, under the null hypothesis (i.e., the SNP was not associated with any phenotype in any study), the test statistic for the meta-analysis followed a chi-square distribution with a DF of 12.

$$\begin{array}{l} \left(-2\right)\sum_{j}In\left(p_{j}\right)\sim \chi_{12}^{2}, \end{array}$$

From which we obtained a meta-analysis *p*-value *p*_*M*_. We adopted the broadly recognized genome-wide significance *p*_*M*_ < 5e−08 as a threshold to declare the significant SNPs.

### Functional annotation

We first pruned the identified SNPs from the pleiotropic meta-analysis to select independent SNPs to represent adjacent loci in high LD for the follow-up studies. LD information for each pair of SNPs was acquired using SNAP (Johnson et al., 2008) and the identified SNPs on each chromosome were aggregated into groups based on pairwise correlation coefficients of LD (i.e., *r*^2^). More specifically, a SNP was merged into a group if it was in high LD (*r*^2^ > 0.5) with any SNP in the group. We selected the SNP with the most significant *p*-value within each group as the proxy. To highlight novel SNPs for the downstream analyses, we examined the identified proxy loci in the Ensembl database and employed GRASP (Leslie et al., 2014) to search GWAS catalog data for previously reported associations. Functional consequences of these identified proxy SNPs were annotated using the Ensembl Variant Effect Predictor (McLaren et al., 2010). To check whether these SNPs had a plausible role in gene expression, we examined whether they were eQTLs or in LD with eQTLs of associated genes. We retrieved information on eQTLs in the gene which the SNP is located in or is flanked by from the GTEx database (Lonsdale et al., 2013) for all available tissues and acquired pairwise LD information using SNAP (Johnson et al., 2008). We examined various regulatory features such as DNase clusters, chromatin interactions, DNA methylation, histone modification from the ENCODE database (The ENCODE Project Consortium, 2012) through the UCSC genome browser. We examined variants in high LD (*r*^2^ > 0.8) with the identified SNPs in non-coding regions for their functional annotation and the presence of chromatin histone marks, hypersensitive DNase elements and protein binding sites from the ENCODE and Roadmap databases by using the HaploReg v3 software (Ward and Kellis, 2012).

### Analysis of age-dependent effects

In order to ensure that the SNPs identified from the time-to-event analysis satisfied the PH assumption and explore their potential age-dependent effects, we checked the model fitting based on the correlation coefficient between transformed survival time and the scaled Schoenfeld residuals (Grambsch and Therneau, 1994). We declared the violation if the *p*-value from the *cox.zph* function in the *survival* R package was < 0.05. Any violation of the PH assumption indicated the existence of age-dependent effects, and for such SNPs we acquired an overall picture of its dynamics of the age-dependent effect by utilizing the *timecox* function in the *timereg* R package (Martinussen and Scheike, 2007), in which we assumed that the counting process started at the age of 40 as data is modeled by a counting process representation in this method. Other covariates such as sex that did not violate the PH assumption were treated as constants and a cluster-based resampling method was used to correct for the family structure. Based on the produced temporal pattern of the dynamic effects, we further performed stratification analyses to investigate the specific effects in each age group.

### Mediation analysis

**Figure 2:**
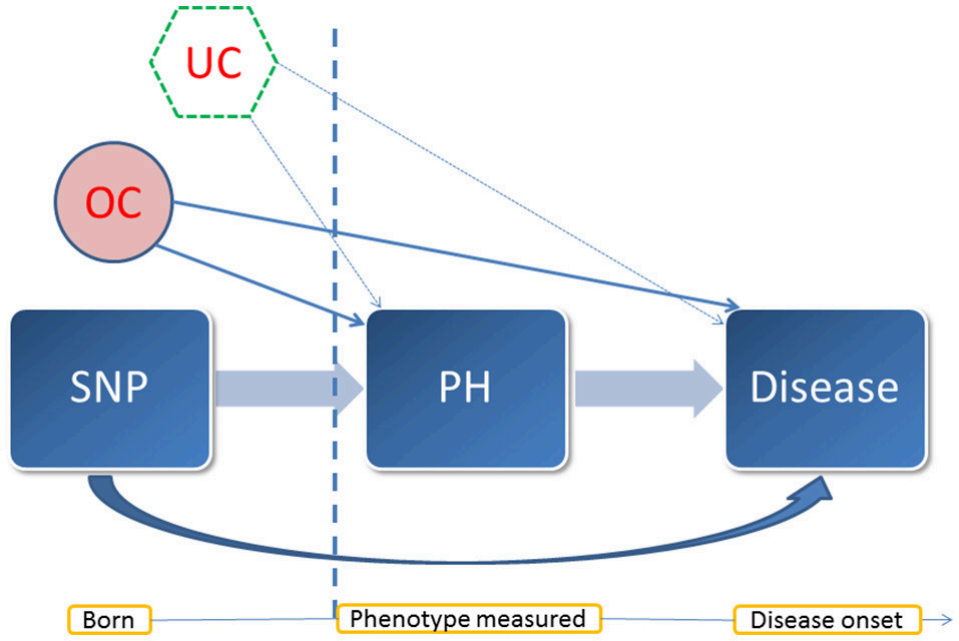
**A diagram of the mediation analysis**. This diagram illustrates the assumed causal relationship between the entities involved in the mediation analysis and their temporal pattern. PH: a quantitative trait treated as a mediator. In this case, it was an endophenotype of interest. OC: observed confounders such as sex and age. UC: potential unmeasured confounders. In the mediation analysis, we assumed that there was no unmeasured confounder behind the baseline measurement of the mediator and the onset of the disease under investigation. Vertical dash line: the time point at the measurement of the mediator.

For the SNPs identified to be associated with both the endophenotypes and complex diseases, we performed mediation analysis to investigate whether the pleiotropic effect is mediated by the endophenotypes (natural indirect effect) or through other unspecified mechanisms (natural direct effect). The rationale of the mediation analysis is illustrated in the causal graph (Figure 2). In the longitudinal data, we chose the measurement at the baseline of an endophenotype as a mediator, which ruled out the possibility of reverse causality for most diseases (except cancer) as the measurements occurred well before the onset of diseases. Due to the Mendelian randomization, in general there should be no confounder other than population stratification between the SNPs and the mediators or diseases. We included age and sex as covariates and further assumed that there were no other unmeasured confounders between the assignment of a mediator at the baseline and a disease. We followed a unified approach proposed by Lange et al. (2012) based on marginal structural models (MSMs) (Robins et al., 2000) to estimate the natural direct and indirect effects. We adopted linear regression for the mediator model, and the Cox PH model for the outcome model. Robust standard errors were obtained using a cluster-based non-parametric bootstrap method, as suggested by Rochon et al. (2014), with 500 replicates to control for the family structure.

## Results

### Pleiotropic meta-analysis identifies seven novel loci

The pre-selection GWAS highlighted 343 SNPs from ARIC that were included in the pleiotropic meta-analysis. If a SNP was not genotyped in the other studies, the imputed genotypes were used if passing the quality control (the detailed information on each of the SNPs is listed in Table S1). The effect sizes and HRs (or ORs) from the association tests in each study are presented in Table S2. Based on the summary statistics, we performed three pleiotropic meta-analyses for: (A) the endophenotypes, (B) the diseases, (C) both. Consequently, out of the 343 SNPs, 148, 11, and 147 SNPs had a *p*-value surpassing the genome-wide significance threshold (5e-08) in each analysis, respectively (Table S1). Four SNPs (rs4506565, rs7901695, rs4132670, and rs8082812) showed significant association in all three analyses, in which the first three are located in *TCF7L2*. After pruning the SNPs based on LD and *p*-values, we ended up with 28, 9, and 28 independent proxy SNPs. After functional annotation and examining the GWAS catalog, we identified three novel SNPs from the analysis of (B) alone: rs7081476 on 10p12.1 located in an intron of *ANKRD26* (*p* = 2.47e-08), rs12420422 on 11q24.1 flanked by *BSX* (~20 kb) and *HSPA8* (~40 kb) (*p* = 5.21e-10) and rs460976 in *TMPRSS2* on 21q22.3 (*p* = 1.37e-08), one novel SNP rs1869717 on 4q31.3 in an intron of *MAML3* from the analysis of (C) alone (*p* = 4.82e-08), two novel SNPs from both analyses of (B) and (C): rs7632505 on 3q21.1 located in an intron of *SEMA5B* (*p* = 8.04e-22 and 7.26e-19), rs4905014 on 14q32.12 located in an intron of *ITPK1* (*p* = 4.93e-16 and 8.85e-15), and rs8082812 on 18p11.22 from all three analyses (*p* = 4.44e-08, 5.71e-64, and 5.01e-67). No novel SNPs were identified from the analysis of (A) alone. Five out of the seven highlighted SNPs are low-frequency variants (1% < MAF < 5%) (The detailed information is given in Table 2).

**Table 2:** **Basic information of the seven identified SNPs with the *p*-values from the pleiotropic meta-analysis and MAFs acorss the five cohorts**.

**SNP ID**	**Chr**	**Position^a^**	**A1**	**A2**	**Associated or flanking gene**	**P^b^**	**P^c^**	**P^d^**	**MAF^e^**
rs7632505	3	123019460	A	G	*SEMA5B* (intron)	1.11e-01	8.04e-22	7.26e-19	ARIC: 34.23%, MESA: 37.63%, FHS1: 16.07 (imp), FHS2: 14.79% (imp), CHS: 23.8% (imp), HRS: 23.5% (imp)
rs1869717	4	139829967	G	C	*MAML3* (intron)	6.91e-04	1.68e-05	4.82e-08	ARIC: 3.19%, FHS1: 3.05%, FHS2: 3.70%, CHS: 8.80% (imp), MESA: NA, HRS: 7.90% (imp)
rs7081476	10	26969741	G	C	*ANKRD26* (intron)	1.76e-01	2.47e-08	4.75e-06	ARIC: 2.12%, MESA: 1.87%, FHS1: 1.81%, FHS2: 2.56%, CHS: 3.1% (imp), HRS: 4.1% (imp)
rs12420422	11	123009573	G	A	*BSX* (~20 kb), *HSPA8* (~40 kb)	8.72e-01	5.21e-10	1.63e-07	ARIC: 2.23%, FHS1: 2.42%, FHS2: 1.67%, MESA: 1.77%, CHS: NA, HRS: 1.74%
rs4905014	14	92945686	G	C	*ITPK1* (intron)	2.96e-02	4.93e-16	8.85e-15	ARIC: 2.85%, MESA: 1.70%, FHS: NA, CHS: NA, HRS: NA
rs8082812	18	8522684	C	A	intergenic	4.44e-08	5.71e-64	5.01e-67	ARIC: 2.41%, MESA: 0.37%, HRS: 2.1% (imp), FHS: NA, CHS: NA
rs460976	21	41463567	G	A	*TMPRSS2* (~1 kb)	9.24e-01	1.37e-08	4.32e-06	ARIC: 2.33%, MESA: 2.16%, FHS1: 0.81%, FHS2: 1.30%, CHS: 2% (imp), HRS: 2.26%

a *The position information is based on the genome assembly version: GRCh38.p5*.

b *The p-values from the pleiotropic meta-analysis of quantitative endophenotypes*.

c *The p-values from the pleiotropic meta-analysis of combined complex diseases*.

d *The p-values from the pleiotropic meta-analysis of both*.

e *The MAFs (A2 is the coding allele) were estimated from the subjects included in the pleiotropic analysis. The expected MAF from IMPUTE2 is reported, denoted by (imp), when a SNP was imputed in that study. NAs imply that the SNP was excluded from that study*.

### Mediated pleiotropic effects of rs7081476 on diseases through endophenotypes

The univariate association tests show that rs7081476 was at least nominally (*p* < 0.05) associated with a variety of endophenotypes and diseases across multiple studies, including BMI (*p* = 2.82e-03 in ARIC), SBP (*p* = 1.51e-02 in ARIC), HDLC (*p* = 6.64e-03 in ARIC), TG (*p* = 3.24e-02 in ARIC), BG (*p* = 1.95e-02 in MESA), hemoglobin A1c (HbA1c) (*p* = 3.44e-03 in MESA), AF (*p* = 2.88e-03 in ARIC), HF (*p* = 8.32e-03 in ARIC), CHD (*p* = 5.50e-10 in ARIC), DM (*p* = 4.89e-02 in ARIC, *p* = 4.78e-04 in MESA), ND (*p* = 9.18e-03 in FHS cohort 2), and stroke (*p* = 7.62e-03 in ARIC, *p* = 2.48e-02 in HRS) (Table 3). The minor allele of this low-frequency variant was associated with elevated BMI, SBP, TG, BG, HbA1c, and reduced HDLC levels. It was associated with increased hazards of AF, HF, CHD, stroke, DM, and reduced hazard of ND consistently across the studies. Based on the GTEx database, rs7081476 is an eQTL of the gene *MASTL* (*p* = 9.2e-06). Furthermore, rs7081476 is in modest LD (the LD structure of CEU estimated from the 1000 Genomes pilot 1) with rs12573313 (*r*^2^ = 0.248) and rs7069690 (*r*^2^ = 0.263), which are eQTLs affecting the expression of multiple genes including *MASTL, LINC00202-1*, etc, in numerous tissues (Table S4). Rs7081476 and multiple variants in high LD (*r*^2^ > 0.8) located in *ANKRD26* were found in strong enhancers in the B-lymphoblastoid cell lines (Table S5). We observed indirect effects, albeit small (< 10% of the total effects), of rs7081476 on stroke, HF and CHD that were partly mediated through BMI, SBP, TG, and HDLC. The mediation analyses also suggest that the effect of rs7081476 on DM was partly mediated through BMI and HDLC in ARIC and predominantly through HbA1c in MESA (Table S3).

**Table 3:** **Nomially significant associations of the seven novel SNPs identified from the univariate analyses in each cohort**.

**SNP ID**	**Study**	**Trait**	**Effect size^a^**	***P*^b^ (< 0.05)**	***P*^c^**
rs7632505	ARIC	BG	0.792	4.83e-03	NA
		TG	1.51	2.61e-02	NA
		HF	0.269	7.30e-08	5.67e-01
		CHD	0.402	2.75e-28	1.5e-05
		Stroke	−0.176	2.14e-02	5.75e-01
	FHS C1	BMI	−1.792	2.17e-02	NA
		TC	−1.695	4.00e-02	NA
	FHS C2	VR	0.929	2.78e-02	NA
	CHS	CHD	0.139	2.87e-02	2.92e-01
	HRS	DM	0.11^d^	1.27e-02	NA
rs1869717	ARIC	DBP	−0.745	1.83e-02	NA
		TC	1.828	6.01e-03	NA
		HF	0.287	1.74e-02	1.75e-01
		CHD	0.516	7.20e-10	5.64e-01
		Stroke	0.412	1.19e-02	3.21e-01
	FHS C1	TG	−12.355	2.05e-02	NA
		Stroke	0.589	1.01e-02	5.69e-01
		Death	0.470	2.59e-04	5.53e-01
	CHS	BMI	−2.451	3.42e-02	NA
		SBP	−4.484	7.35e-04	NA
		HDLC	4.837	4.70e-03	NA
		TG	−7.805	9.66e-03	NA
		FEV1	−0.095	9.45e-03	NA
	HRS	BG	1.727	2.39e-03	NA
		DM	0.19^d^	4.33e-02	NA
rs7081476	ARIC	BMI	2.51	2.82e-03	NA
		SBP	1.626	1.51e-02	NA
		HDLC	−3.54	6.64e-03	NA
		TG	4.68	3.24e-02	NA
		DM	0.0489	4.89e-02	4.95e-01
		AF	0.412	2.88e-03	8.36e-02
		HF	0.363	8.32e-03	6.16e-01
		CHD	0.582	5.50e-10	7.32e-01
		Stroke	0.492	7.62e-03	5.76e-01
	FHS C2	ND	−1.884	9.18e-03	8.01e-01
	MESA	BG	3.42	1.95e-02	NA
		HbA1c	0.18	3.44e-03	NA
		DM	0.626	4.78e-04	4.95e-01
	CHS	HF	0.437	5.07e-03	1.41e-01
	HRS	Stroke	0.286^d^	2.48e-02	NA
		Death	0.26	1.88e-02	2.53e-01
rs12420422	ARIC	Death	0.266	1.65e-02	1.75e-01
		AF	0.331	1.96e-02	7.60e-01
		HF	0.425	3.18e-03	5.96e-01
		CHD	0.775	5.20e-17	8.41e-01
	FHS C1	DBP	2.384	1.62e-02	NA
rs4905014	ARIC	HC	0.376	9.78e-04	NA
		Stroke	1.166	3.75e-21	6.8e-03
	MESA	DBP	−1.925	2.55e-02	NA
		HbA1c	0.17	1.44e-02	NA
		HF	1.029	2.58e-02	5.65e-01
rs8082812	ARIC	BG	4.042	2.63e-07	NA
		DBP	−0.827	1.07e-02	NA
		HDLC	−2.993	8.12e-03	NA
		TC	2.778	4.64e-05	NA
		Death	0.279	1.46e-03	4.49e-01
		AF	0.305	5.99e-03	2.21e-01
		HF	0.538	1.66e-07	4.86e-01
		CHD	0.848	3.37e-37	1.43e-02
		Stroke	1.226	3.40e-41	2.91e-03
	MESA	BMI	8.018	3.83e-02	NA
rs460976	ARIC	HF	0.468	2.99e-04	1.39e-01
		CHD	0.714	1.13e-16	2.92e-01
	FHS C2	TG	−9.927	3.08e-02	NA
	HRS	DM	0.22	4.14e-02	NA

a Log-transformation (100^*^log(.)) was performed on the original measurements for some of the endophenotypes (Please refer to Table 1 for the details). For the time-to-event traits, this is log(HR).

b *The p-value of the association with an endophenotype or a disease from that cohort*.

c *The p-value from the cox.zph function for checking the PH assumption. A p-value < 0.05 leads to rejection of the assumption. It is not available for quantitative traits*.

d *This is an OR as prevalence was used instead of time-to-event traits in HRS*.

### Rs12420422 and rs460976 are associated with heart related diseases

Rs12420422 was nominally associated with the hazards of AF (*p* = 1.96e-02), HF (*p* = 3.18e-03), CHD (*p* = 5.20e-17), and death (*p* = 1.65e-02) in ARIC. A missense variant rs2276348 located in *CLMP* was found to be in high LD (*r*^2^ = 0.91) with rs12420422 (Table S5). Multiple variants in high LD (*r*^2^ > 0.8) with rs12420422 were found within strong enhancers in multiple cell lines (Table S5). The minor allele of rs12420422 increased the hazards of AF, HF, CHD, and death. Rs460976, a downstream gene variant of *TMPRSS2* located in a copy number variation (CNV) (esv3647063), was associated with HF (*p* = 2.99e-04) and CHD (*p* = 1.13e-16) in ARIC (Table 3). Rs460976 is in modest LD with rs7280422 (*r*^2^ = 0.124), which is an eQTL associated with the expression of *MX1* in transformed fibroblasts, testis and tibial artery tissues, and rs11910387 (*r*^2^ = 0.141), which is an eQTL associated with the expression of *MX2* in adipose-subcutaneous and skin (Table S4).

### Rs7632505 is associated with BG and TG, and has an age-dependent effect on CHD

**Figure 3:**
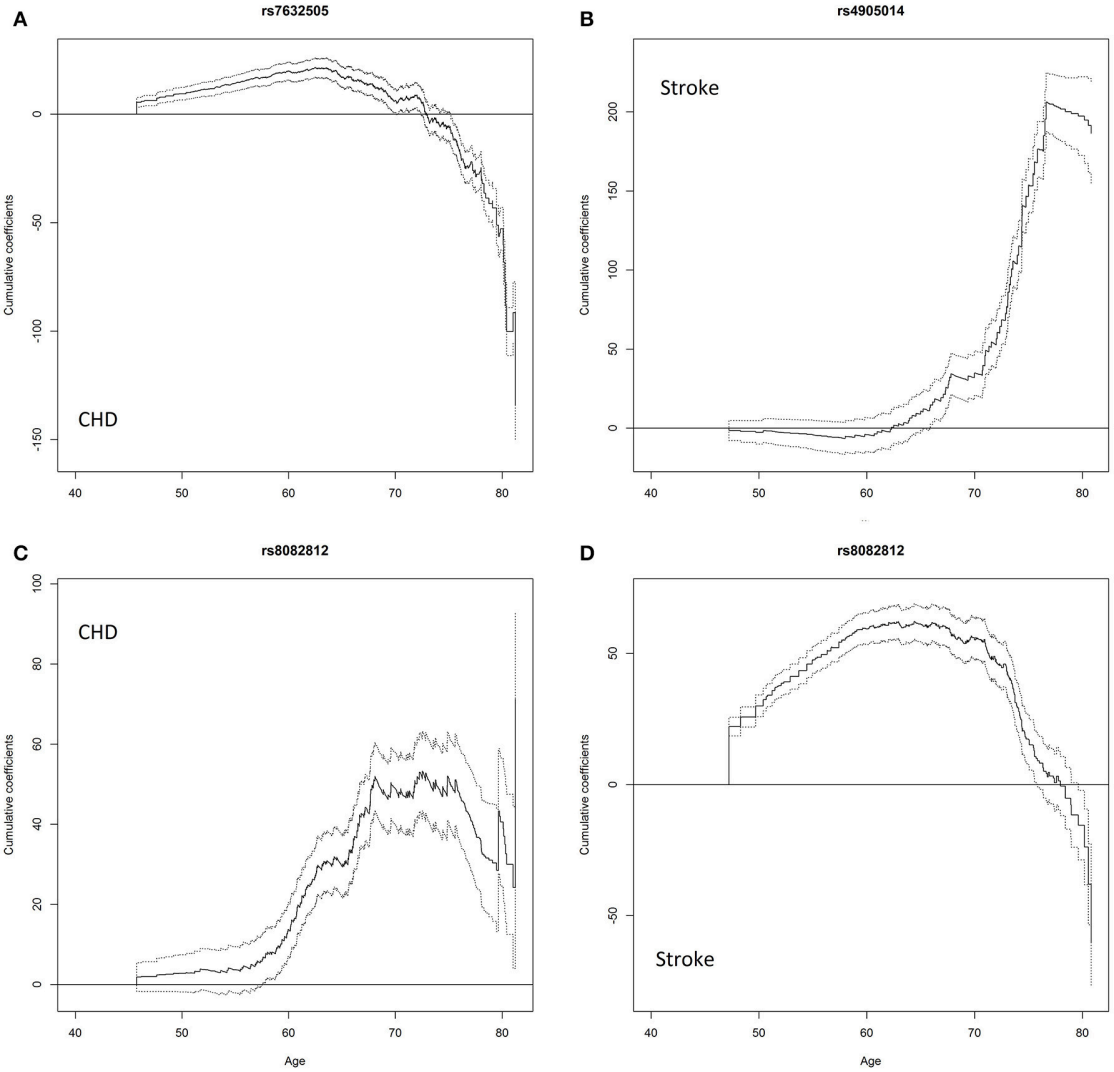
**Cumulative age-dependent effects of the minor allele on the onset of diseases estimated using the *timecox* function that fits a proportional hazards model allowing time-varying effects of the SNPs**. The solid lines are cumulative time-varying regression coefficient estimates with respect to age. The dash lines are 95% pointwise confidence bands. **(A)** effect of rs7632505 on CHD, **(B)** effect of rs4905014 on stroke, **(C)** effect of rs8082812 on CHD, **(D)** effect of rs8082812 on stroke.

The univariate association tests indicate that rs7632505, a non-sense-mediated mRNA decay transcript variant, was nominally associated with BG (*p* = 4.83e-03), TG (*p* = 2.61e-02), HF (*p* = 7.30e-08), and stroke (*p* = 2.14e-02) in ARIC, BMI (*p* = 2.17e-02), TC (*p* = 4.00e-02), and VR (*p* = 2.78e-02) in FHS, CHD in ARIC and MESA (*p* = 2.75e-28 and 2.87e-02) and DM in HRS (*p* = 1.27e-02) (Table 2). Rs7632505 is in modest LD with rs72974405 (*r*^2^ = 0.342) and rs9829771 (*r*^2^ = 0.283), which are eQTLs associated with the expression of *PDIA5* in tibial artery tissues (Table S4). The test of the PH assumption suggests that the effect on the hazard of CHD was far from proportional (*p* = 1.5e-05 from *cox.zph*) (Table 3). The age-dependent analysis using the *timecox* function shows that the effect on the hazard of CHD dropped dramatically after the age of ~70 (Figure 3A). The survival curves from the Kaplan–Meier (K-M) estimator were consistent (Figure S7). The stratified analysis suggests that the minor allele increased the hazard of CHD before the age of ~74 (Age 45–74: HR = 1.498, *p* = 2.92e-27) while decreases it after that (Age 75–85: HR = 0.769, *p* = 0.101). No mediation effects through BG or TG were observed on CHD, HF, or stroke (Table S3).

### Rs4905014 has an age-dependent effect on stroke

Nominally significant association of rs4905014 was observed with HC (*p* = 9.78e-04) and stroke (*p* = 3.75e-21) in ARIC, and with DBP (*p* = 2.55e-02), HbA1c (*p* = 1.44e-02), and HF (*p* = 2.58e-02) in MESA (Table 3). The minor allele of this low-frequency variant was associated with elevated HC, HbA1c levels, increased hazards of stroke and HF, and decreased DBP levels. The PH test suggests that the hazards of CHD were not proportional with respect to the SNP (*p* = 6.8e-03 from *cox.zph*). The time-dependent analysis using the *timecox* function shows that the effect on stroke was age-dependent (Figure 3B), and the detrimental effect on the hazard of stroke remained almost imperceptible until age 62 but accelerated substantially after the age of ~70. The survival curves from the K-M estimator were consistent (Figure S7). The stratified analysis shows that the HRs before and after age 62 were 1.8408 (*p* = 0.077) and 3.748 (*p* = 2.38e-25), respectively. The annotation using the Ensembl Variant Effect Predictor shows that rs4905014 is located in a promoter flanking region of a transcript of *ITPK1* in multiple cell types (ENSR00001854796). Rs4905014 is within a DNase hypersensitive site in the neuroblastoma cell line (SK-N-SH_RA) and multiple variants in high LD (*r*^2^ > 0.8) were found in strong enhancers in multiple cell lines from the ENCODE database (Table S5). The mediation analysis suggests that no mediation effect through HC was observed on the hazard of stroke (Table S3).

### Rs8082812 and rs1869717 are associated with both endophenotypes and diseases

Rs8082812 was found to be at least nominally associated with BG (*p* = 2.63e-07), DBP (1.07e-02), HDLC (*p* = 8.12e-03), TC (4.64e-05), the hazards of death (*p* = 1.46e-03), AF (*p* = 5.99e-03), HF (*p* = 1.66e-07), CHD (*p* = 3.37e-37), and stroke (*p* = 3.40e-41) in ARIC and BMI (*p* = 3.83e-02) in MESA (Table 2). The minor allele of this SNP was associated with elevated BG and TC levels, increased hazards of AF, CHD, stroke, HF and death, and decreased DBP and HDLC levels. The test of the PH assumption suggests that the effect of the SNP on the hazard of stroke was not proportional (*p* = 2.91e-03 from *cox.zph*) and the time-dependent analysis shows that the effects on the hazards of CHD and stroke were age-dependent and exhibited different temporal patterns (Figures 3C,D). The survival curves from the K-M estimator were consistent (Figure S7). The effect on CHD remained modest before age 60, increased dramatically between ages 60–70 and leveled off subsequently. The stratified analysis shows that the effect on stroke was much larger and more significant before age 73 than after that (HR = 8.23, *p* = 3.47e-54 vs. HR = 1.78, *p* = 0.229). We further noticed that one copy of minor allele had an HR of 7.27 (*p* = 1.01e-51) while two copies had no significant effect (HR = 2.39, *p* = 5.82e-02) on stroke. Functional annotation suggests that rs8082812 is located in a DNase hypersensitive site in the fibroblasts and urothelial cell lines (Table S5) and close to a copy number variation (~1.4 kb) (Ensembl ID: esv3641717). The mediation analysis suggests that the effect of rs8082812 on the hazard of HF was partly mediated through HDLC, while the effect on CHD was partly mediated through BG and TC (Table S3). Rs1869717, located in *MAML3* and an enhancer region (ENSR00002016330), was at least nominally associated with DBP (*p* = 1.83e-02), TC (*p* = 6.01e-03), HF (*p* = 1.74e-02), CHD (*p* = 7.20e-10) and stroke (1.19e-02) in ARIC, stroke (*p* = 1.01e-02) and death (*p* = 2.59e-04) in FHS cohort 1, BMI (*p* = 3.42e-02), SBP (*p* = 7.35e-04), HDLC (*p* = 4.70e-03), and FEV1 (*p* = 9.45e-03) in CHS, BG (*p* = 2.39e-03) and DM (4.33e-02) in HRS, and TG in FHS cohort 1 and CHS (*p* = 2.05e-02 and *p* = 9.66e-03) (Table 3). The minor allele of this SNP increased the hazards of HF, CHD, stroke, DM and death, but exhibited heterogeneous effects on the endophenotypes. No indirect effects were observed on any of the diseases in ARIC (Table S3).

## Discussion

With the recent discovery of a large number of genes and variants influencing endophenotypes and diseases simultaneously (Sivakumaran et al., 2011), pleiotropy has been attracting a lot of interest. A fundamental hypothesis in gerontology is that delaying biological aging process, which is assumed to lead to physical dysfunction and to be implicated in multiple age-related diseases through common underlying mechanisms (i.e., pleiotropic effects), would simultaneously delay the onset and progression of these diseases (Kaeberlein et al., 2015). For example, chronic diseases of aging including CVDs, cancer and ND share pathological links through infection and inflammation (Franceschi, 2007; Larbi et al., 2008; Finch, 2010, 2012). Searching for pleiotropic genetic variants helps validate these substantive hypotheses and pinpoints mechanisms that could be biologically plausible for the most basic processes associated with aging in human. By performing phenome-wide (more than 20 phenotypes or age-related diseases) association analyses followed by pleiotropic meta-analyses with five large-scale longitudinal cohorts, we identified seven novel SNPs that affect multiple endophenotypes and age-related diseases simultaneously. Five out of the seven SNPs are low-frequency variants which might be the reason why they were overlooked in the previous GWAS. It is worth pointing out that almost all these low-frequency variants show detrimental effects, the excess of which may suggest the polygenic inheritance in these diseases (Chan et al., 2014).

Our results suggest that the genome-wide significance of rs7081476 is achieved by aggregating its association with a wide spectrum of diseases across different cohorts. Rs7081476 is located in an intron of *ANKRD26*, which is involved in hematopoiesis, glucose homeostasis, fat cell differentiation, fatty acid and lipid metabolism, among the other processes (Fei et al., 2011; Raciti et al., 2011; Cesar et al., 2015). Mutations in *ANKRD26* or the promoter region have been reported to cause autosomal dominant non-syndromic thrombocytopenia-2 (THC2) (low platelet count) (Noris et al., 2011; Pippucci et al., 2011; Al Daama et al., 2013). Platelets represent an important connection between inflammation and development of CVDs (Willoughby et al., 2002; Wagner and Burger, 2003), which may justify the association with a variety of CVDs in our findings. A mouse model also confirmed that partial inactivation of the *ANKRD26* gene caused the development of extreme obesity, insulin resistance and gigantism (Bera et al., 2008), which is in line with our findings of DM and BMI. We further show that the effects of rs7081476 on stroke, HF, CHD, and DM were mediated through several endophenotypes among which BMI had the most significant indirect effect. It has been pointed out that controlling the expression of *ANKRD26* would affect megakaryopoiesis and platelet production, perhaps by inducing apoptosis (Pippucci et al., 2011) or MAPK pathway hyperactivation (Bluteau et al., 2014). Interestingly, elevated platelet count may correlate with obesity (Samocha-Bonet et al., 2008). On the other hand, the neighboring *MASTL* kinase, of which rs7081476 is a direct eQTL, is important for cell cycle regulation and is implicated in normal thrombopoiesis and association with THC2 (Di Paola and Johnson, 2011). This evidence indicates that rs7081476 may be involved in regulatory activity of the genes in THC2 locus functionally related to the hematopoiesis.

Rs12420422 is an intergenic variant flanked by the genes *BSX* (~28 kb) (encoding a brain-specific homeobox protein) and *HSPA8* (~48 kb) (encoding a heat shock protein) with an average MAF of ~2% estimated from the five cohorts. While associated with the CVDs (most significantly with CHD) and mortality, no association was detected with any of the endophenotypes, which may suggest that the effects on the CVDs are irrelevant of the pathway related to the endophenotypes under investigation. Members of the stress-inducible heat-shock protein family, including *HSPA8*, are associated with the development of atherosclerosis and CHD/coronary artery disease (CAD) (Latchman, 2001; Mehta et al., 2005; He et al., 2010; Zhang et al., 2014). *HSPA8* is also involved in immune response, the regulation of hematopoiesis and autophagy among the other processes (Zou et al., 2011; Stricher et al., 2013).

Rs460976 associated with HF and CHD is a low-frequency downstream gene variant located in an intergenic region flanked by *TMPRSS2* and *MX1*. Similar to rs12420422, no association was detected with the selected endophenotypes, suggesting that the effects on HF and CHD are probably through other mechanisms. One potential mechanism is through immunological effects. Indeed, *TMPRSS2* has an important role in influenza virus and other viral respiratory infections whereas *MX1* participates in the cellular antiviral response. CVDs can be associated with influenza-like illness (Kwok et al., 2015) probably through immune mechanisms of atherosclerosis (Frostegård, 2013).

Rs7632505 has recently been reported to be associated with thromboangiitis obliterans (OR = 29.47, *p* < 1e-04), a chronic peripheral vascular occlusive disease in a Xinjiang Uyghur Population (Shi et al., 2016). The dynamic effect of rs7632505 on CHD may be explained by the inhibitory effect of *SEMA5B* on angiogenesis which is related to aging process (Lähteenvuo and Rosenzweig, 2012) and is purported to be involved in atherosclerosis and other CVDs (Khurana et al., 2005; Deveza et al., 2012). *SEMA5B* is a member of the phylogenetically conserved class 5 semaphorins that all contain a characteristic Sema domain along with thrombospondin repeats (Adams et al., 1996) and is functionally important for angiogenesis (Iruela-Arispe et al., 1999; Chen et al., 2000; Grundmann et al., 2013). The moderate expression of *SEMA5B* is detected in multiple tissues including heart and brain (Nagase et al., 2000). Besides, *SEMA5B* functions as a guidance cue and is implicated in Ca^2+^-mediated growth cone collapse (To et al., 2007). On the other hand, rs7632505 may associate with the expression of the neighboring *PDIA5* gene through LD with the eQTLs and *PDIA5* is involved in the endoplasmic reticulum (ER) redox homeostasis. In addition, regulatory activity of the *PDIA5* gene is required for normal hematopoiesis (Gieger et al., 2011; Bielczyk-Maczyńska et al., 2014).

We observed that the effect of rs4905014 on the onset of stroke grew rapidly after age ~65, suggesting that it may have an increasing etiologic effect on stroke for the elderly. Its effects on stroke and HF were not mediated through HC, DBP, or HbA1c. The related gene *ITPK1* is involved in inositol signaling pathways which regulate the conductance of calcium-activated chloride channels (Chamberlain et al., 2007; Saiardi and Cockcroft, 2008). The enzyme is associated with neural tube defects (Majerus et al., 2010; Guan et al., 2014). A study using an expression probe array and rt-PCR has found that *ITPK1* expression in peripheral blood shows a significant correlation with a CAD index (Sinnaeve et al., 2009).

Rs8082812, located in a DNase hypersensitive site and surrounded by regulatory regions, is adjacent to (~300 b) an enhancer (ENSR00001899253) active in multiple cell lines, ~1.4 kb from a CNV (esv3641717) and ~2.4 kb from a CTCF binding site (ENSR00000667587). *RAB12* (~87 kb) and *PTPRM* (~116 kb) are the closest protein coding genes. *RAB12* is involved in protein transport, degradation of transferrin receptor and autophagy (Matsui and Fukuda, 2013). Maintenance of cellular iron homeostasis is indispensable in the management of diseases and aging, and thus relates to mortality (Altamura and Muckenthaler, 2009; Weinberg, 2010). Inhibition or over-activation of autophagy might be associated with various CVDs (Salabei and Conklin, 2013). *PTPRM* is required for neurite outgrowth and axonal migration by regulating adhesion through the classical cadherins (Oblander and Brady-Kalnay, 2010). This gene contributes to potassium channels gene regulation in adult cardiac myocytes by cell-cell contact (Hershman and Levitan, 2000). Also, *PTPRM* may play a role in angiogenesis (Sommer et al., 1997). Its heterogeneous dynamic patterns of age-dependent effects on the hazards of stroke and CHD may be partly explained by its effects on a broad spectrum of endophenotypes.

Rs1869717 is located in *MAML3* (Mastermind Like Transcriptional Coactivator 3), which is a transcriptional coactivator for *NOTCH* proteins (Wu et al., 2002). A previous GWAS finds that another SNP (rs1531070) in *MAML3* is significantly (*p* = 4.99e-12) associated with congenital heart malformation in Han Chinese (Hu et al., 2013). Another genome-wide meta-analysis identifies a locus near this gene that is associated with protein intake (Tanaka et al., 2013).

To sum up, all of the identified pleiotropic SNPs are either within a gene or next to regulatory regions, highlighting the probability of their involvement in gene expression or various regulatory activities. A previous report discovers that disease-associated variants are frequently located in strong enhancer elements and patterns of enhancer activity are strongly correlated with patterns of nearest-gene expression (Ernst et al., 2011). Our results showing that most of the identified variants are associated with tissue-specific strong enhancers, DNase clusters or missense variants are consistent with the previous finding. On the other hand, we further discovered the age-dependent effects of some SNPs on CHD and stroke, confirming the previous findings that the effects of variants on the complex diseases in post-reproductive period could be age-dependent (Kulminski, 2013; Kulminski et al., 2015b; Yashin et al., 2015). It seems that their pleiotropic effects result from their diverse involvement in different underlying mechanisms potentially related to nervous, cardiovascular and immune system functions including axon guidance and angiogenesis (*SEMA5B, PTPRM*), ion channels (*SEMA5B, PTPRM, ITPK1*), stress resistance, inflammation and immunity (*HSPA8, TMPRSS2*/*MX1*), and hematopoiesis (*ANKRD26, HSPA8*). On the other hand, some of the SNPs such as rs7081476 and rs8082812 are related to lipids or blood related metabolites. As these metabolites have critical influence on CVDs, DM and thus eventually mortality, the strong association of these SNPs with age-related diseases is mediated, at least in part, through their effects on the metabolites, which can be classified as mediated pleiotropic effects. Interestingly, rs7632505 and rs8082812 with age-dependent effects of minor alleles are linked to genes (*SEMA5B, PTPRM*) involved in axonal migration and angiogenesis which are age-dependent processes.

The power advantage of the pleiotropic analysis can be demonstrated by rs8082812 for which the *p*-value from the pleiotropic meta-analysis was more significant than any from the univariate analysis. It can be seen from the results of the pleiotropic analysis for the 343 SNPs that the omnibus test based on the O'Brien's test (O'Brien, 1984) increases the statistical power when the pleiotropic effect of a SNP is heterogeneous and opposite to the correlation between the statistics from the univariate analysis. Nevertheless, it may not be the optimal solution to detecting a SNP that affects two correlated phenotypes in the same direction through independent paths. Many extensions to the general omnibus tests have been proposed by assigning different weights for phenotypes (Xu et al., 2003; Zhu et al., 2015). In general, no methods are optimal in all situations, and the choice of these methods depends on the assumptions we make and the sort of pleiotropic effect we aim to detect.

Although the large-scale data sets under investigation are widely recognized and documented, there still exist some limitations. The definitions and criteria of measurements of the complex diseases and the targeted populations vary in the five cohorts. Moreover, some SNPs are not available in other cohorts as different genotyping chips are adopted in these studies. Imputation did not come to a rescue for harmonizing missing data of low-frequency variants as the imputation quality is low. Because of these, the replication of a significant association in other cohorts is not straightforward. The lack of some replications may also suggest that the effects of age-related loci are more sensitive to environment such as age as many of the identified SNPs have age-dependent effects. In addition, in terms of the statistical power of the omnibus test, the inclusion of many endophenotypes reduces the chance to detect the SNPs that are only associated with a small proportion of phenotypes. Methods with adaptive weights on different phenotypes might identify more signals in this case. Another problem is that although we assumed that there were no other unmeasured confounders than the included covariates underlying the endophenotypes at the baseline and the onset of the complex diseases, it is still possible that these confounders exist, and in this case, the estimated mediation effect would be biased (Pearl, 2000). For example, we observed an indirect effect but no direct effect of rs7081476 on DM that was mediated through HbA1c. However, the no-confounder assumption was probably violated for HbA1c and DM as it has been shown that the association of HbA1c with DM may be explained by its biomarker for the presence and severity of hyperglycemia (Lyons and Basu, 2012).

In conclusion, we identified seven novel loci that exhibit pleiotropic effects on a variety of endophenotypes and age-related diseases using five large-scale longitudinal cohorts. The enrichment of identified pleiotropic variants involved in immune system and inflammation supports the hypothesis of shared pathological role of infection and inflammation in chronic age-related diseases. The findings indicating the potential regulatory involvement of these genetic variants in a diversity of functions contribute to the understanding of the shared mechanisms underlying comorbidity and the relationship between anthropometry, lipid- and blood-related traits, CVDs, DM, and NDs. Our results suggest that the effects on the risk of CVDs often follow different age-dependent patterns and are probably through angiogenesis. These findings are valuable for guiding follow-up analyses and provide evidence for further scrutinizing the molecular mechanisms and implication of the relevant genes in the age-related diseases.

## Author contributions

Design of the work (AK, LH), acquired the phenotype data (OB, MD, AIY, SU, MK, KA, AY), acquired the genotype data (AIY, SU, MK, KA, AY), genotype quality control and other manipulations (YL), analyzed the data (LH, YK, AK, YL), interpreted the results (AK, LH, IK, EL), and wrote the paper (LH, AK, IK, EL, YK).

## Funding

The research reported in this paper was supported by Grants No P01 AG043352 and R01 AG047310 from the National Institute on Aging (NIA). The funders had no role in study design, data collection and analysis, decision to publish, or preparation of the manuscript. The content is solely the responsibility of the authors and does not necessarily represent the official views of the National Institutes of Health (NIH).

### Conflict of interest statement

The authors declare that the research was conducted in the absence of any commercial or financial relationships that could be construed as a potential conflict of interest.

## Supplementary material

Table S1 **The basic information of the 343 SNPs selected from the GWAS with the ARIC dataset (SNP ID, chromosome, position, alleles, MAF, imputation) and *p*-values from the three pleiotropic analyses in each study**. The genome-wide significant (*p* < 5e-08) SNPs identified from the pleiotropic analysis of endo-phenotypes, complex diseases and both are highlighted in red, blue and green, respectively.

Table S2 **Endophenotype- and disease-specific summary statistics [effect sizes, HRs (or ORs), and *p*-values] from the univariate analyses in each study of the 343 SNPs selected from the GWAS with the ARIC dataset**. The numbers of SNPs in FHS, CHS, and HRS are less than 343 as some SNPs were excluded due to poor imputation quality in these studies.

Table S3 **Results from the mediation analyses for the five identified SNPs that are associated with both endophenotypes and diseases**. HR, Hazard ratio; CI, 95% confidence interval; P, *p*-value from the bootstrap method; Direct, the effect of the SNP on the disease directly or through other unmeasured mechanisms; Indirect, the effect of the SNP on the disease mediated through the endo-phenotype; Total, the total effect of the SNP on the disease.

Table S4 **The information extracted from the GTEx database (as of Sep 2016) of the eQTLs in the nearby or associated genes in moderate or modest LD with the identified SNPs**. Rs7069690 and rs12573313 are in LD with the identified SNP rs7081476, which is an eQTL *per se*. Rs7280422 and rs11910387 are in LD with rs460976. Rs72974405 and rs9829771 are in LD with rs7632505. Gencode ID: Ensembl ID; Effect size, *P*-value: the effect size and *p*-value of the association test of the gene expression; Tissue: the tissue in which the gene expression is measured.

Table S5 **The regulatory characterization extracted from the ENCODE and Roadmap databases using HaploReg v3 of the seven identified SNPs together with the variants in high LD (*r*^2^ > 0.8)**. AFR, AMR, ASN, EUR: The MAFs of this variant in African, American, Asian and European populations; Promoter_ENCODE, Enhancer_ENCODE, Promoter_Roadmap, Enhancer_Roadmap: The promoter or enhancer information including the promoter or enhancer type and the cell line of this variant in the ENCODE and Roadmap databases.

Figure S1 **A heatmap of the correlation matrix of the summary statistics estimated from the univariate association analysis in ARIC**.

Figure S2 **A heatmap of the correlation matrix of the summary statistics estimated from the univariate association analysis in FHS cohort 1**.

Figure S3 **A heatmap of the correlation matrix of the summary statistics estimated from the univariate association analysis in FHS cohort 2**.

Figure S4 **A heatmap of the correlation matrix of the summary statistics estimated from the univariate association analysis in MESA**. SBP was removed in the follow-up pleiotropic meta-analysis as it was highly correlated with (*r* > 0.7) DBP and PP (brightly yellow in the figure).

Figure S5 **A heatmap of the correlation matrix of the summary statistics estimated from the univariate association analysis in CHS**. FVC was removed in the follow-up pleiotropic meta-analysis as it was highly correlated with FEV1 (*r* > 0.7) (brightly yellow in the figure).

Figure S6 **A heatmap of the correlation matrix of the summary statistics estimated from the univariate association analysis in HRS**.

Figure S7 **A plot of K-M survival curves for the SNPs rs7632505, rs4905014, and rs8082812, which exhibit age-dependent effects on CHD or stroke**.
