# Corrigendum: Pleiotropic Meta-Analyses of Longitudinal Studies Discover Novel Genetic Variants Associated with Age-Related Diseases

**DOI:** 10.3389/fgene.2017.00226

**Published:** 2018-01-12

**Authors:** Liang He, Yelena Kernogitski, Irina Kulminskaya, Yury Loika, Konstantin G. Arbeev, Elena Loiko, Olivia Bagley, Matt Duan, Arseniy Yashkin, Svetlana V. Ukraintseva, Mikhail Kovtun, Anatoliy I. Yashin, Alexander M. Kulminski

**Affiliations:** Biodemography of Aging Research Unit, Social Science Research Institute, Duke University, Durham, NC, United States

**Keywords:** pleiotropic analysis, age-related traits, CVDs, genetic association study, mediation analysis, age-dependent effects

In the original article, we included a supplementary material “Presentation 1.PDF.” The original purpose of this supplementary material is to provide readers with more explanation of the marginal structural model (MSM) we used for mediation analysis. However, we made some mistakes in the mathematical expression in the supplementary material which can be misleading. For more details about the model, it would be better for readers to directly refer to the three cited articles in which the model is proposed. Thus, we think that this supplementary material is inaccurate and redundant, and should be removed. Note that this supplementary material was only for illustrative purpose and does not affect any results in the article. So removing it will not change anything else in the article. In addition, we should have added in the subsection “Pleiotropic Meta-Analysis” the following statement “We checked the multivariate normality assumption for these summary statistics under the null hypothesis using the Henze-Zirkler test (Henze and Zirkler, 1990), and found no evidence of violation of the assumption.” This statement ensures the validity of the omnibus test for our analyses.

The authors apologize for this error and state that this does not change the scientific conclusions of the article in any way.

The original article has been updated.

## Conflict of interest statement

The authors declare that the research was conducted in the absence of any commercial or financial relationships that could be construed as a potential conflict of interest.
