# Supplementary figures and images for: Pleiotropic Meta-Analyses of Longitudinal Studies Discover Novel Genetic Variants Associated with Age-Related Diseases
Source: Front Genet. 2016 Oct 13;7:179. doi: 10.3389/fgene.2016.00179 (PMC5061751; doi:10.3389/fgene.2016.00179)

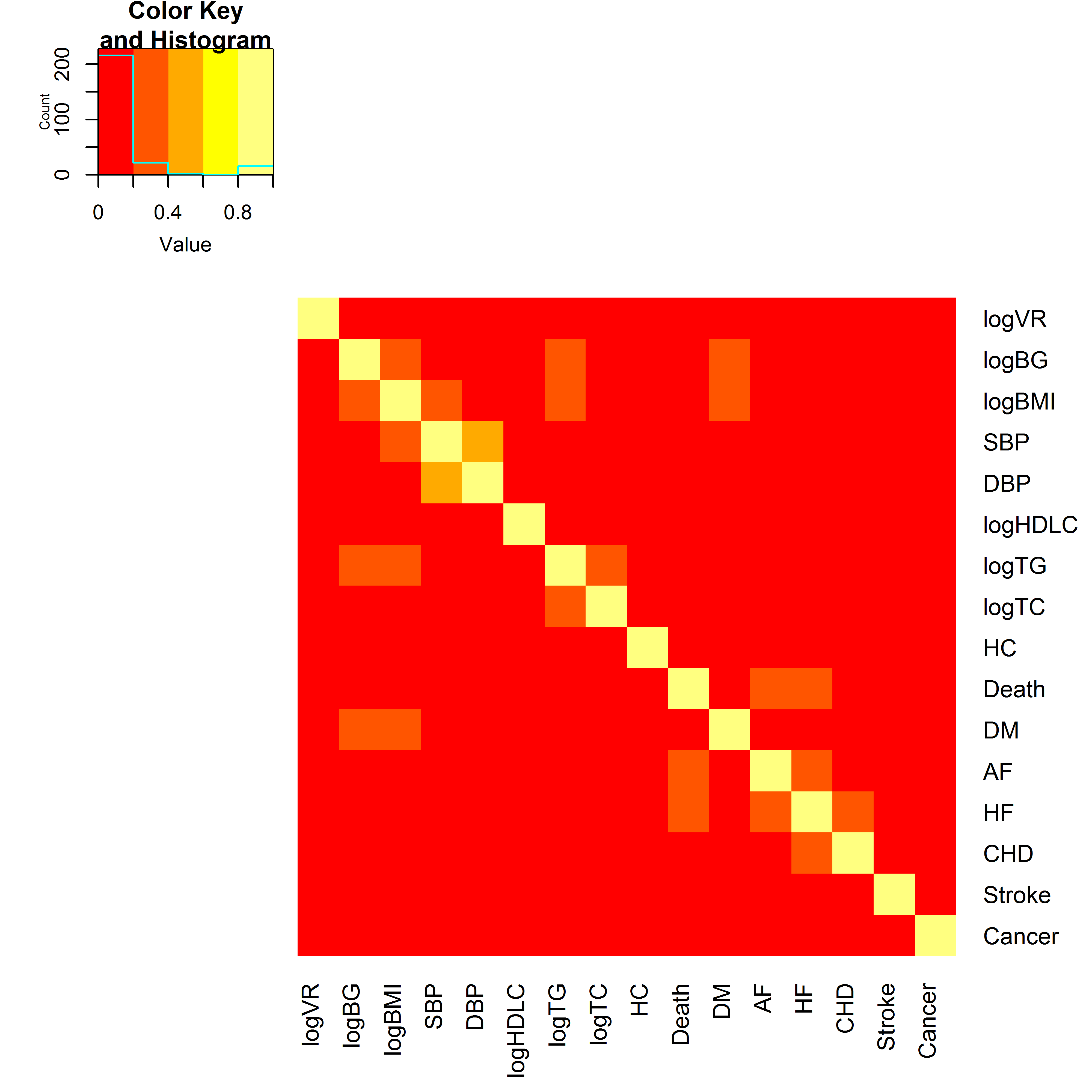

Supplement: Figure S1 — A heatmap of the correlation matrix of the summary statistics estimated from the univariate association analysis in ARIC. [file Image1.PNG]

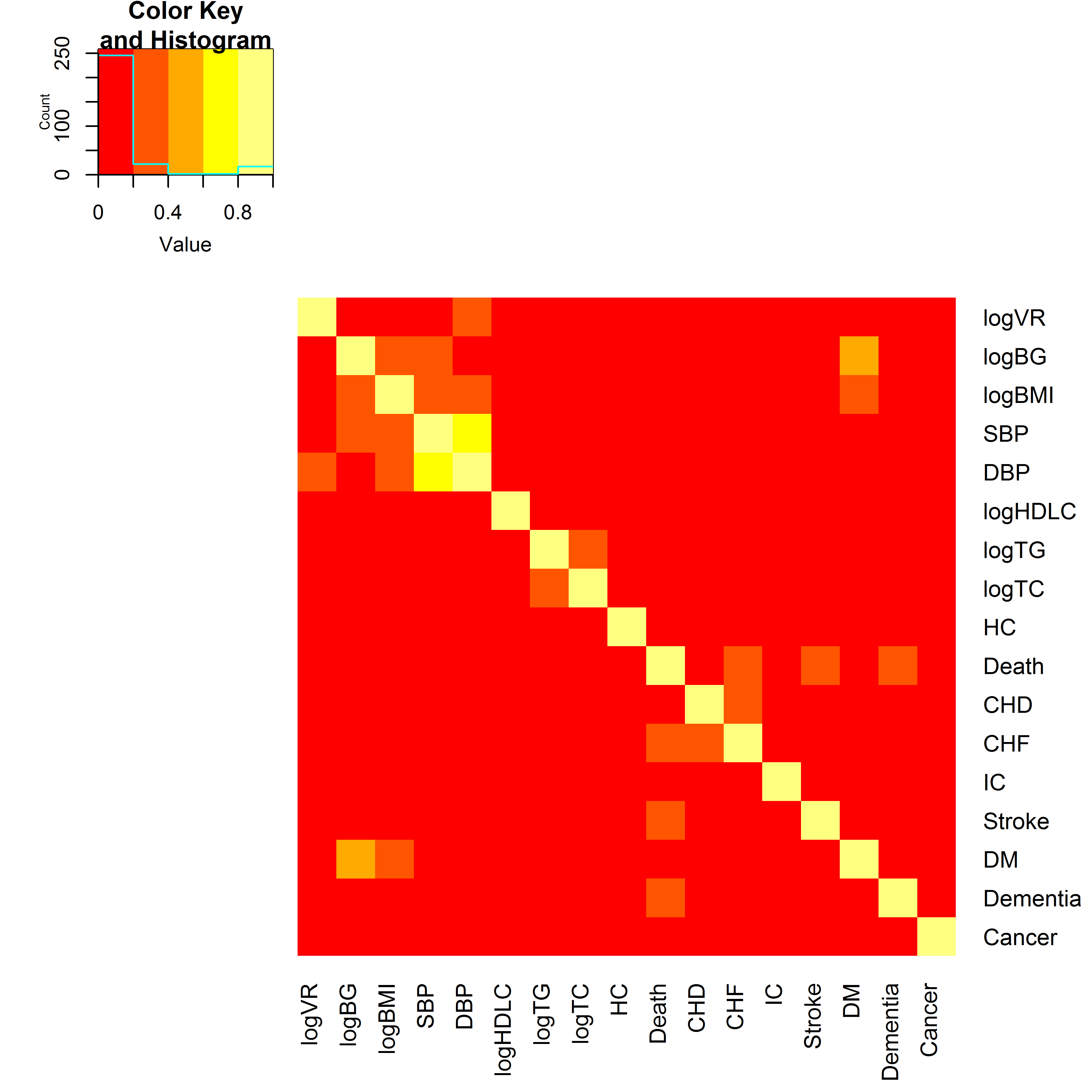

Supplement: Figure S2 — A heatmap of the correlation matrix of the summary statistics estimated from the univariate association analysis in FHS cohort 1. [file Image2.PNG]

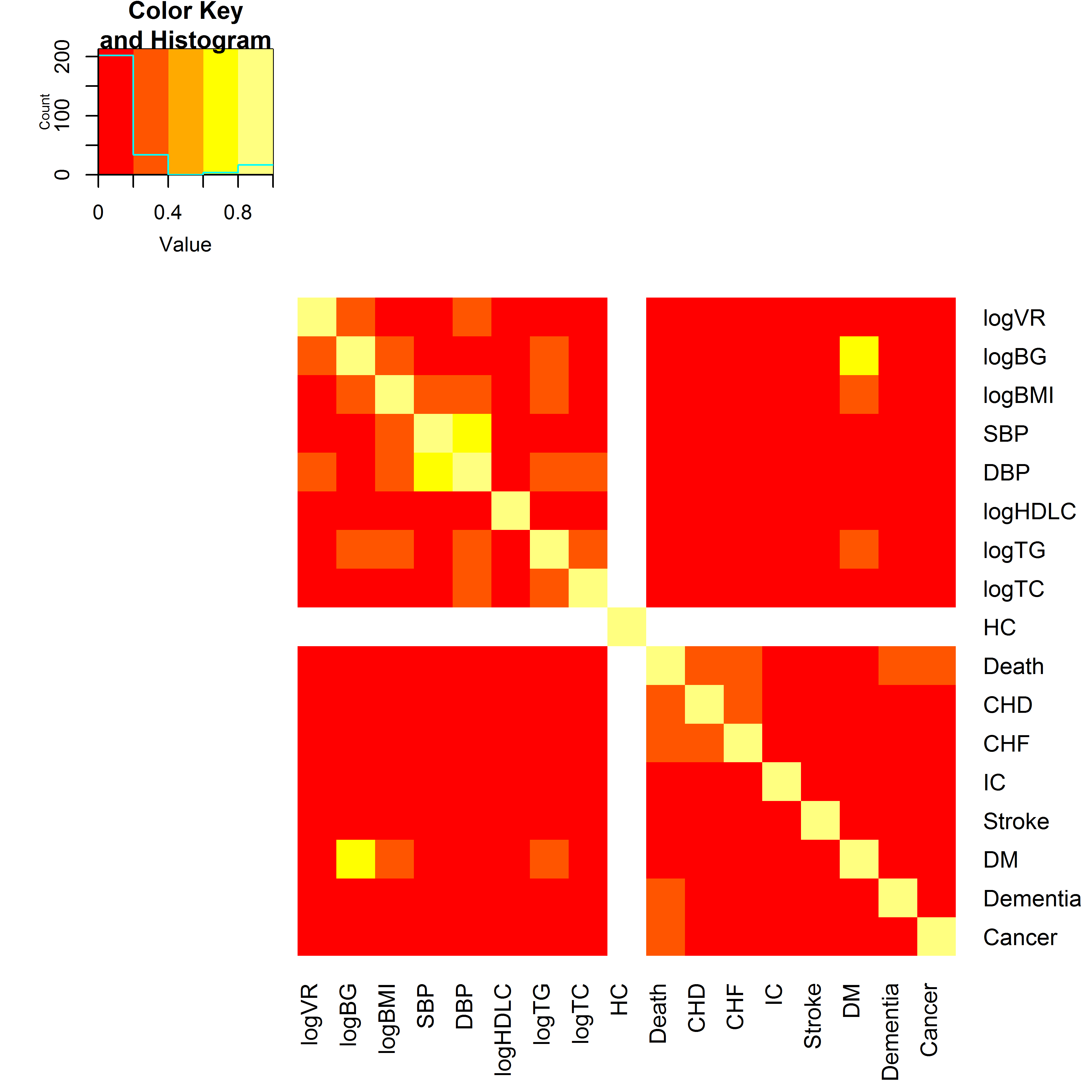

Supplement: Figure S3 — A heatmap of the correlation matrix of the summary statistics estimated from the univariate association analysis in FHS cohort 2. [file Image3.PNG]

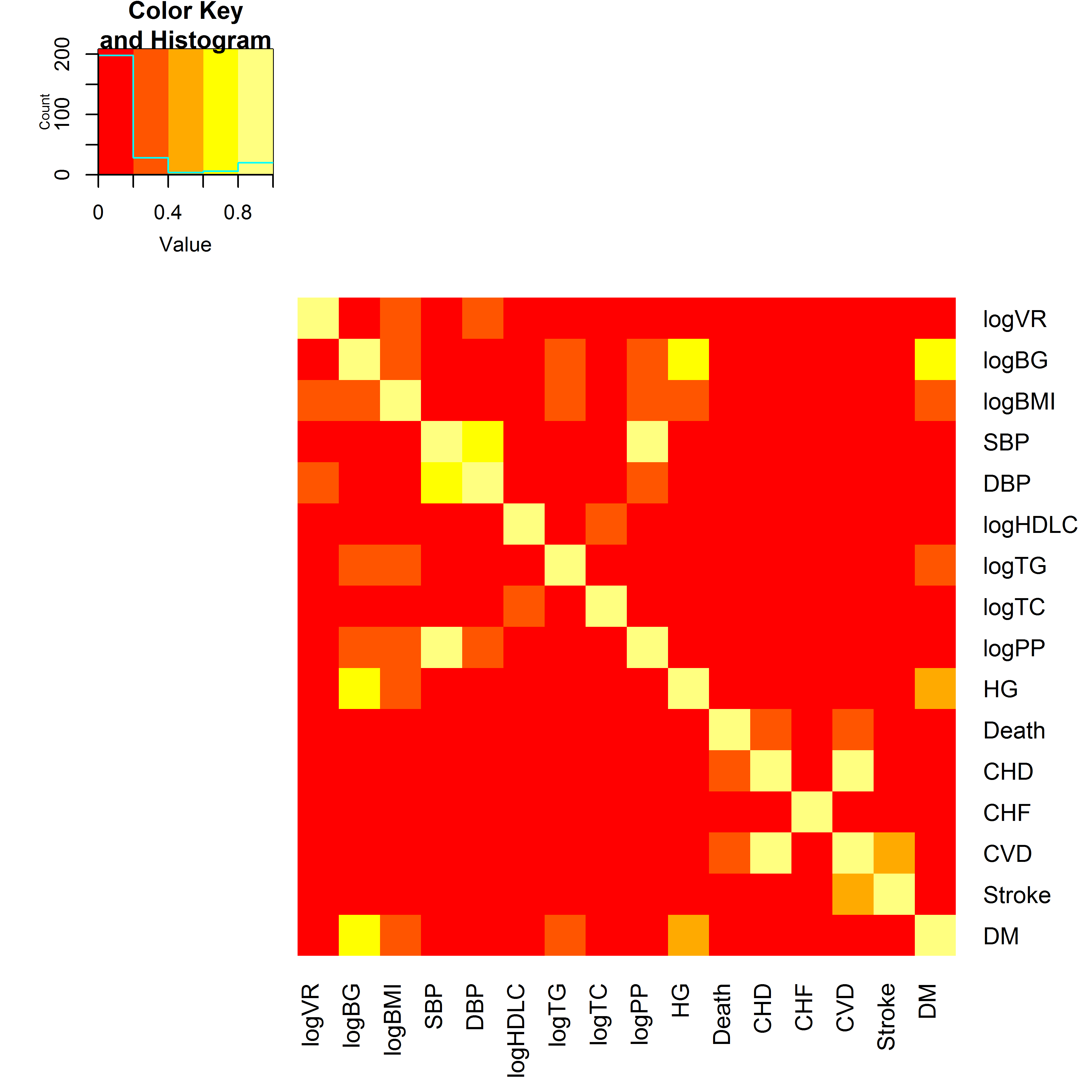

Supplement: Figure S4 — A heatmap of the correlation matrix of the summary statistics estimated from the univariate association analysis in MESA. SBP was removed in the follow-up pleiotropic meta-analysis as it was highly correlated with (r > 0.7) DBP and PP (brightly yellow in the figure). [file Image4.PNG]

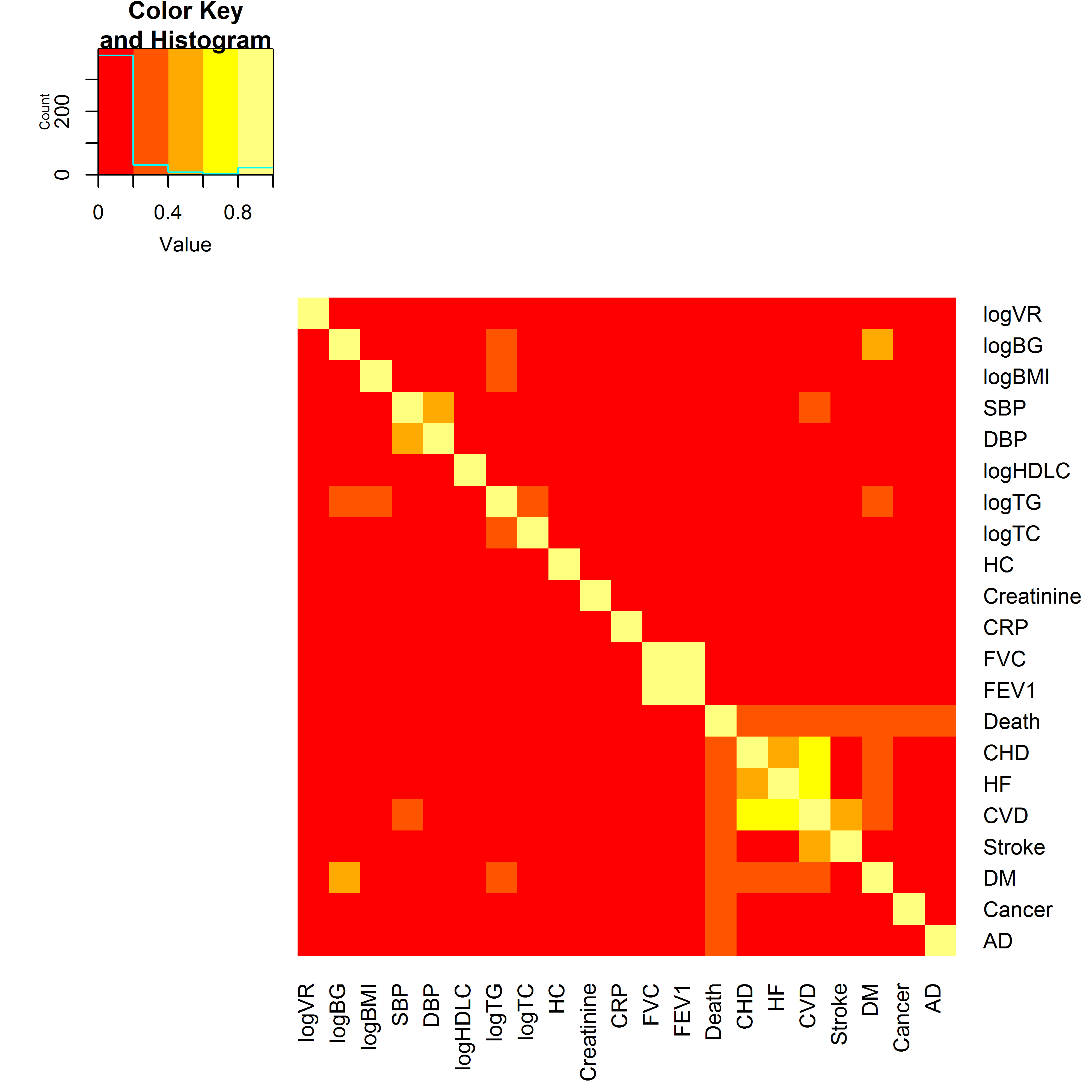

Supplement: Figure S5 — A heatmap of the correlation matrix of the summary statistics estimated from the univariate association analysis in CHS. FVC was removed in the follow-up pleiotropic meta-analysis as it was highly correlated with FEV1 (r > 0.7) (brightly yellow in the figure). [file Image5.PNG]

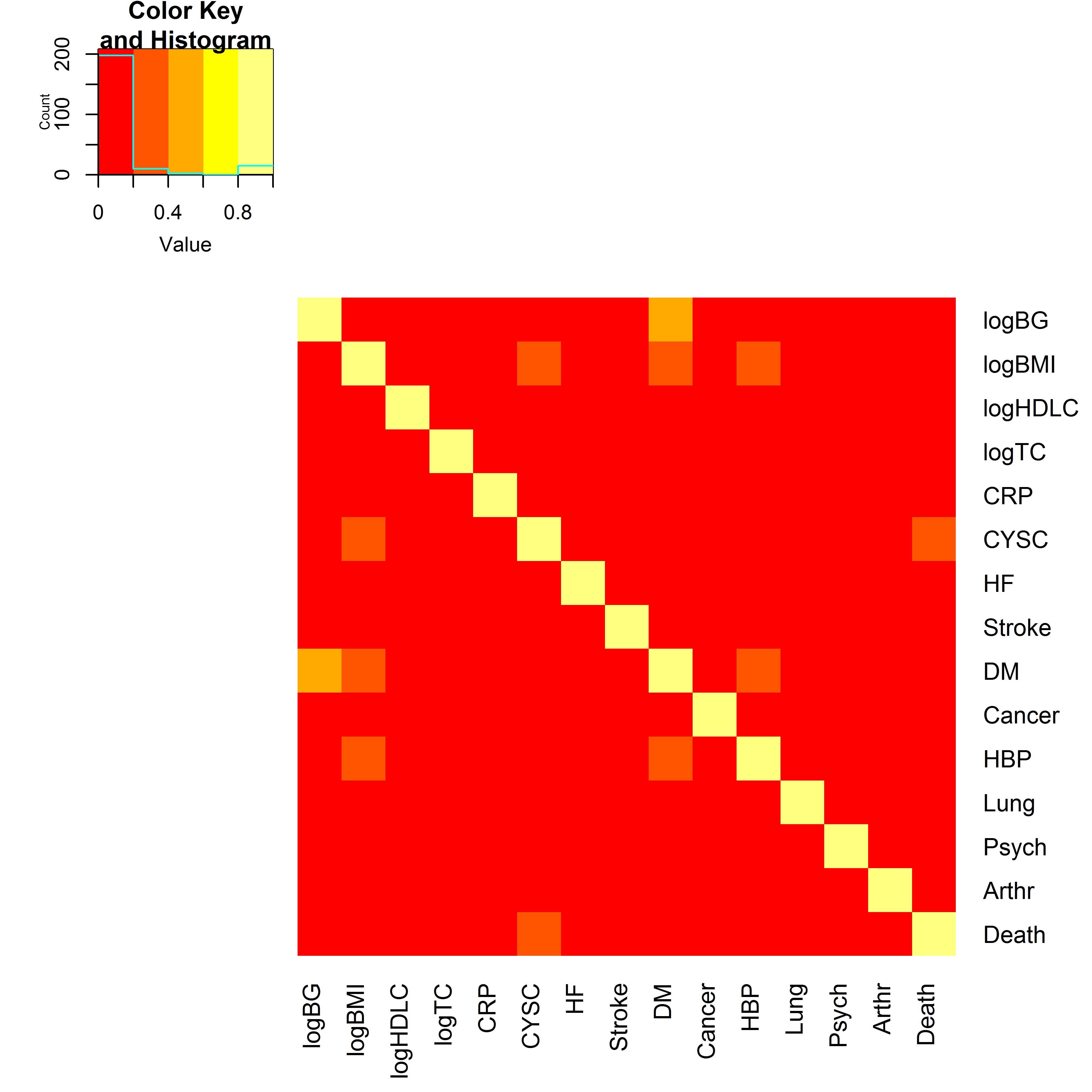

Supplement: Figure S6 — A heatmap of the correlation matrix of the summary statistics estimated from the univariate association analysis in HRS. [file Image6.PNG]

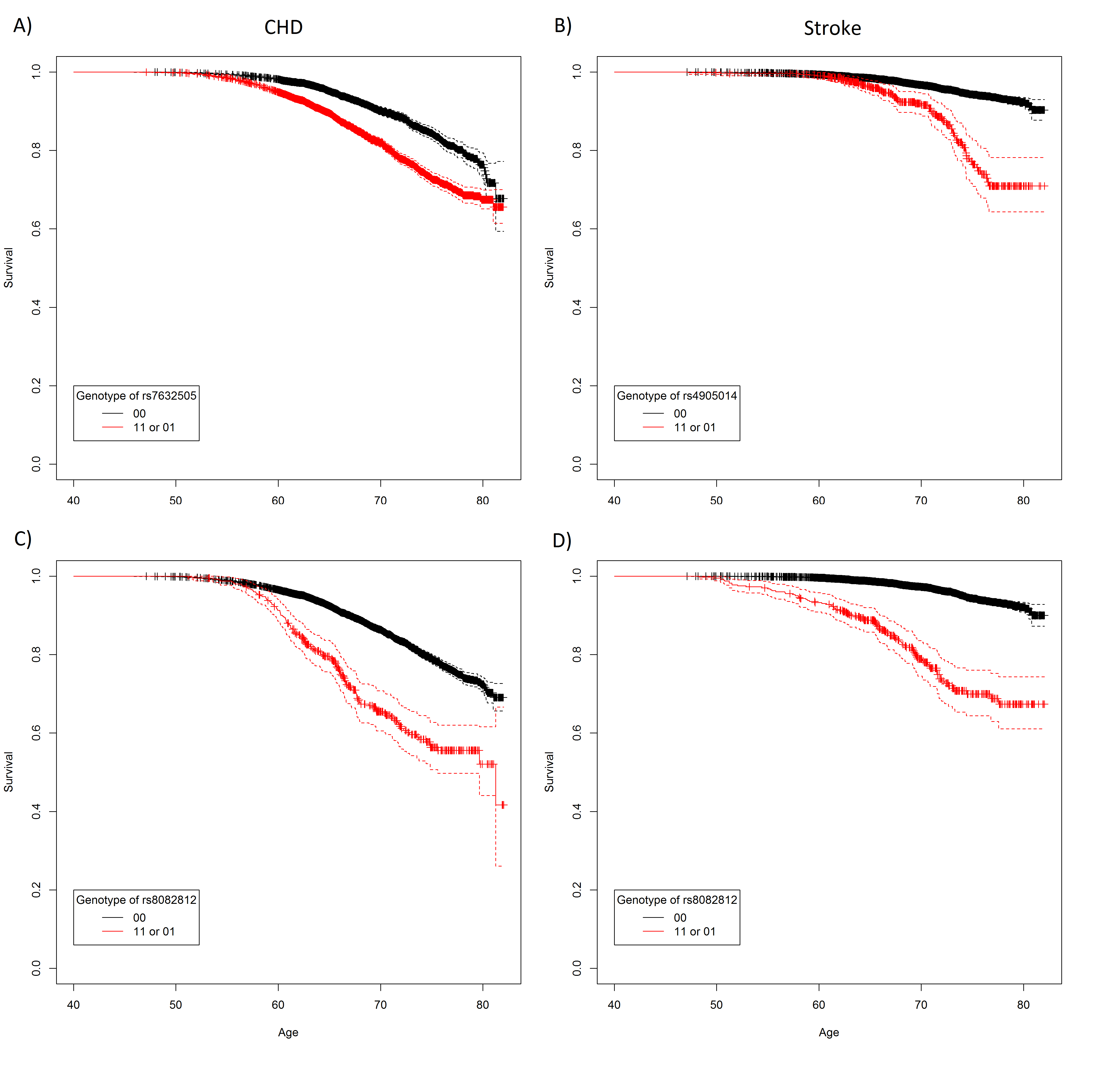

Supplement: Figure S7 — A plot of K-M survival curves for the SNPs rs7632505, rs4905014, and rs8082812, which exhibit age-dependent effects on CHD or stroke. [file Image7.PNG]
